# Brain partial volume correction with point spreading function reconstruction in high-resolution digital PET: comparison with an MR-based method in FDG imaging

**DOI:** 10.1007/s12149-022-01753-5

**Published:** 2022-05-26

**Authors:** Masanobu Ibaraki, Keisuke Matsubara, Yuki Shinohara, Miho Shidahara, Kaoru Sato, Hiroyuki Yamamoto, Toshibumi Kinoshita

**Affiliations:** 1 Department of Radiology and Nuclear Medicine, Akita Research Institute of Brain and Blood Vessels, 6-10 Senshu-Kubota Machi, Akita, 010-0874 Japan; 2 Department of Management Science and Engineering, Faculty of System Science and Technology, Akita Prefectural University, Yurihonjo, Japan; 3 Department of Quantum Science and Energy Engineering, Graduate School of Engineering, Tohoku University, Sendai, Japan

**Keywords:** Brain, Digital PET, Partial volume correction, PSF reconstruction

## Abstract

**Objective:**

In quantitative positron emission tomography (PET) of the brain, partial volume effect due mainly to the finite spatial resolution of the PET scanner (> 3 mm full width at half maximum [FWHM]) is a primary source of error in the measurement of tracer uptake, especially in small structures such as the cerebral cortex (typically < 3 mm thickness). The aim of this study was to evaluate the partial volume correction (PVC) performance of point spread function-incorporated reconstruction (PSF reconstruction) in combination with the latest digital PET scanner. This evaluation was performed through direct comparisons with magnetic resonance imaging (MR)-based PVC (used as a reference method) in a human brain study.

**Methods:**

Ten healthy subjects underwent brain ^18^F-FDG PET (30-min acquisition) on a digital PET/CT system (Siemens Biograph Vision, 3.5-mm FWHM scanner resolution at the center of the field of view) and anatomical T1-weighted MR imaging for MR-based PVC. PSF reconstruction was applied with a wide range of iterations (4 to 256; 5 subsets). FDG uptake in the cerebral cortex was evaluated using the standardized uptake value ratio (SUVR) and compared between PSF reconstruction and MR-based PVC.

**Results:**

Cortical structures were visualized by PSF reconstruction with several tens of iterations and were anatomically well matched with the MR-derived cortical segments. Higher numbers of iterations resulted in higher cortical SUVRs, which approached those of MR-based PVC (1.76), although even with the maximum number of iterations they were still smaller by 16% (1.47), corresponding to approximately 1.5-mm FWHM of the effective spatial resolution.

**Conclusion:**

With the latest digital PET scanner, PSF reconstruction can be used as a PVC technique in brain PET, albeit with suboptimal resolution recovery. A relative advantage of PSF reconstruction is that it can be applied not only to cerebral cortical regions, but also to various small structures such as small brain nuclei that are hardly visualized on anatomical T1-weighted imaging, and thus hardly recovered by MR-based PVC.

**Supplementary Information:**

The online version contains supplementary material available at 10.1007/s12149-022-01753-5.

## Introduction

Positron emission tomography (PET) is an imaging modality that can be used to estimate biological functions in the brain, such as perfusion, metabolism, neuroreceptor density, and amyloid/tau protein accumulation. In small structures, the accuracy of such estimates is limited by partial volume effect (PVE), mainly due to the finite spatial resolution of the PET scanner [1, 2]. This means that reconstructed PET images are spatially blurred compared with the true spatial distribution of radioactivity, resulting in erroneous measurements of tracer uptake. For example, cerebral cortical thickness is typically 2–3 mm [3], whereas the intrinsic spatial resolution of clinical PET scanners is generally 4–6 mm at full width at half maximum (FWHM), and even in the latest generation of digital PET scanners using silicon photomultiplier (SiPM)-based detectors it is only 3–4 mm [4–6]. Furthermore, the reported spatial resolution of brain-dedicated high-resolution PET scanners is still 2–3 mm [7], indicating that even under the best conditions currently possible, some PVE-induced underestimation of cerebral cortical uptake is inevitable.

In the field of brain PET, a number of partial volume correction (PVC) methods using anatomical magnetic resonance (MR) images have been proposed [8–10]. The methods are commonly termed post-reconstruction MR-based PVC, and their advantages include ease of implementation and good recovery performance. In recent years, the geometric transfer matrix (GTM) [11] and its voxel-level extensions [9, 12] have become standard strategies in brain PET research. Although these methods are inherently superior to other MR-based PVC methods in terms of more accurate quantification [12–14], a common limitation is that they assume spatial uniformity of tracer uptake within volumes of interest (VOIs) and calculate the spill-in/spill-out effects only between the VOIs (i.e., inter-regional PVC); thus in MR-based PVC, different VOI settings provide different results [15–17].

Another possible approach for PVC is point spread function (PSF) reconstruction, the ordered-subsets expectation–maximization (OSEM) reconstruction incorporating spatial blurring effects in the system matrix [18–21]. PSF reconstruction is now clinically available on most commercial PET scanners and is advantageous over MR-based PVC in that anatomical MR images are not needed. However, because of its slow convergence speed, PSF reconstruction requires a large number of iterations to achieve high resolution recovery (exceeding the intrinsic scanner resolution), which unfortunately also results in increased image noise [21]. In typical clinical PET systems, it is unusual to apply a large number of iterations because of image noise amplification, and PSF reconstruction is not generally used as the PVC method in brain PET. Therefore, the PVC performance and characteristics of PSF reconstruction with a large number of iterations are not fully understood.

The aim of this study was to evaluate the PVC performance of PSF reconstruction especially for measurements of tracer uptake in the cerebral cortex. The latest digital PET scanners have superior intrinsic spatial resolution, superior timing resolution (time of flight [TOF] resolution), and higher sensitivity than conventional PET scanners [4–6], meaning that higher effective spatial resolution with lower image noise can be achieved. Our hypothesis was that the latest digital PET combined with PSF reconstruction with a high number of iterations would be an effective PVC strategy in brain PET. We therefore conducted an ^18^F-FDG PET study on healthy humans making comparisons with an MR-based PVC reference method.

## Materials and methods

### Subjects

**Fig. 1:**
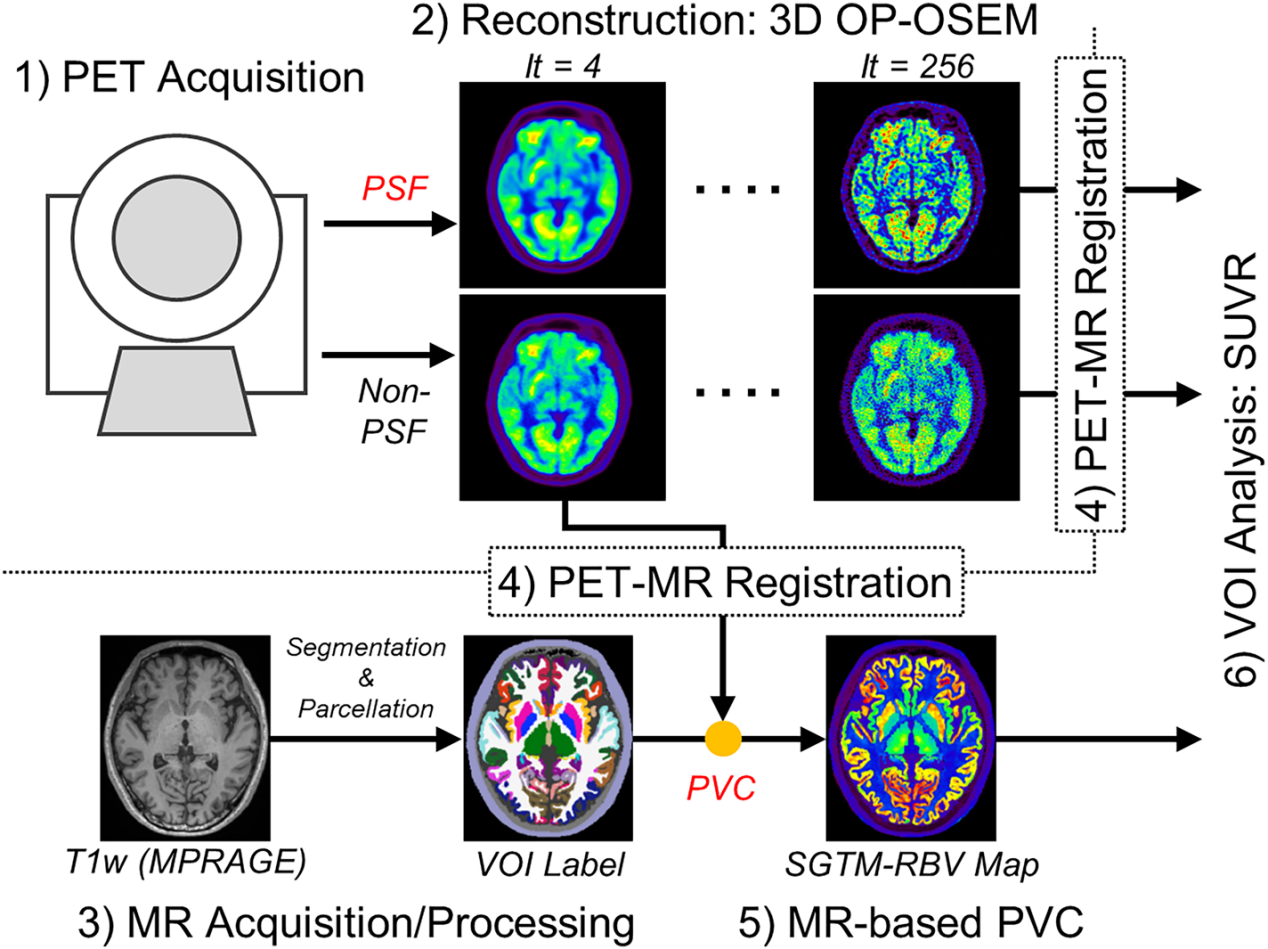
Data acquisition and image processing: (1) PET acquisition, (2) PET image reconstruction, (3) MR acquisition and VOI generation, (4) PET–MR registration, (5) MR-based PVC, and (6) VOI analysis

Ten healthy subjects (5 men and 5 women; 56 ± 5 y) were recruited to the study. All subjects were determined as healthy according to their medical history and MR imaging findings (anatomical images and MR angiography of the brain). Written informed consent was obtained from each subject before the examinations. This study was approved by the Ethics Committee of the Research Institute for Brain and Blood Vessels-Akita (reference number: 18–10), and was performed in accordance with the Declaration of Helsinki. A flowchart summarizing the data acquisition and processing is shown in Fig. 1.

1. Positron emission tomography: acquisition
  A Biograph Vision SiPM PET/CT system (Siemens Healthineers) [5, 22] was used. The transverse and axial spatial resolution of this system are 3.6- and 3.5-mm FWHM, respectively, at a 1-cm offset from the center of the field of view (FOV) [5]. The subjects fasted for at least 4 h prior to scanning, then received an intravenous injection of ^18^F-FDG (229 ± 16 MBq; as per the clinical protocol in our institute). They were asked to keep their eyes open and remain in a resting condition during scanning. To minimize head movement during scanning, the subject’s head was fixed using pads and a Velcro band tightened around the head and head holder. A 30-min PET list-mode acquisition was started 30 min after the ^18^F-FDG injection, which resulted in sufficiently high measurements of 950 ± 185 million coincidences (true plus scatter). For attenuation correction, a standard low-dose CT scan (120 kV, 100 mAs) was acquired.
2. Positron emission tomography: image reconstruction
  The list-mode data were reconstructed into single static images (30-min duration) using the e7-tools off-line reconstruction package (Siemens Healthineers). Images were reconstructed using a 3D ordinary Poisson ordered-subset expectation–maximization (OP-OSEM) algorithm with TOF information and with or without PSF modeling, referred to as PSF reconstruction and non-PSF reconstruction, respectively [18, 22]. The iterations were varied over a wide range: 4, 8, 16, 32, 64, 128, and 256 (5 subsets). The vendor-recommended setting is three to four iterations for whole-body ^18^F-FDG acquisitions [23, 24]. Random coincidences, detector sensitivity, radioactive decay, dead-time count losses, scatter coincidences (single scatter simulation), and attenuation were corrected during the reconstruction. The resulting PET images had a 440 × 440 × 159 matrix (0.8 × 0.8 × 1.6 mm voxel size; post-reconstruction zoom factor 2). Post-reconstruction filtering was not applied. Non-PSF reconstruction images with four iterations were further input into the MR-based PVC.
3. Magnetic resonance imaging: acquisition and volume-of-interest generation
  An isotropic T1-weighted image (MPRAGE; voxel size, 0.8 mm; matrix size, 320 × 320 × 208; sagittal slices; repetition time/echo time/inversion time, 2300/3/900 ms; flip angle, 9 deg; acquisition time, 7 min 21 s) was acquired using a 3-T scanner (MAGNETOM Skyra, Siemens Healthineers). The T1-weighted imaging was processed with FreeSurfer version 7.1.0 (https://surfer.nmr.mgh.harvard.edu/; *recon-all* with high-resolution option) to obtain subcortical segmentations and cortical labels [25]. Extracerebral segments (CSF, skull, and other remaining head tissues) were also included [15], resulting in a total of 114 regions for the whole-brain. We refer to this VOI set as *VOI*_*Full*_, and used it in the MR-based PVC and VOI analysis.
4. Image registration between PET and MR
  The reconstructed PET images were registered to individual T1-weighted volumes (non-brain tissues were stripped) using normalized mutual information criteria (SPM tool [*spm_coreg*]). Subsequent MR-based PVC and VOI analysis were performed in native MR space (Fig. 1). For MR-based PVC maps, PVC-optimization registration, an iterative framework of PET–MR image registration and MR-based PVC, was applied [26].
5. MR-based partial volume correction
  MR-based PVC was performed on the non-PSF reconstruction images with four iterations (Fig. 1). The PVC method used was a region-based voxel-wise correction (RBV) in which the mean VOI values are first estimated using the GTM, and the Yang correction is then applied to calculate the PVC maps [12]. In the implementation used in this study (in-house Matlab routines), a symmetric GTM was used [27].
  The MR-based PVC is expected to yield accurate resolution recovery if the requisite assumptions are fulfilled; in this respect, the importance of the VOI definition used has been emphasized [15, 16]. To evaluate variations in MR-based PVC due to different VOI settings, we investigated five different settings (Supplementary Table 1): *VOI*_*Full*_ as a reference condition, and *VOI*_*BG*_, *VOI*_*Outer*_, *VOI*_*Full*+*WM*_, and *VOI*_*Full*+*WM2*_. In the *VOI*_*BG*_ and *VOI*_*Outer*_ settings, instead of the extracerebral segments in *VOI*_*Full*_, a non-brain background region (all voxels outside the brain) and a 15-mm shell surrounding the outer surface of the brain were used, respectively. In the *VOI*_*Full*+*WM*_ and *VOI*_*Full*+*WM2*_ settings, the subcortical white matter (SCWM) region in each hemisphere in each subject was further segmented into smaller regions according to FreeSurfer’s white matter parcellation. Further details are provided in Supplementary Information.
  The PSF setting, i.e., the FWHM of the Gaussian kernel, should be matched to the effective spatial resolution of the input PET images [28, 29]. Phantom experiments were performed to estimate the effective spatial resolution of the input images (non-PSF with 4 iterations) [30], and these are described in the Supplementary Information. Based on the phantom results (Supplementary Fig. 2), an isotropic 4.0-mm FWHM was assumed for the MR-based PVC. In addition, a 3.5-mm FWHM corresponding to the intrinsic scanner resolution at the FOV center [5] was also applied to investigate the impact of small differences in FWHM.
6. Volume-of-interest analysis
  Cortical FDG uptake was measured using standardized uptake value (SUV) and its ratio (SUVR) with the cerebellar cortex as a reference region. Mean values were calculated for ten cortical regions (frontal, parietal, precuneus, occipital, lateral temporal, medial temporal, anterior cingulate, posterior cingulate, sensory motor, and insula) and SCWM. A whole cerebral cortex VOI consisting of all cortical regions was also defined. Intra-region coefficient of variation (CoV; standard deviation in VOI divided by the mean in % unit) was also presented as a surrogate index of image noise. CoVs were firstly measured for the smallest anatomical segments defined by the VOI_Full+WM2_ setting, and then composite measures for ten cortical regions and SCWM were calculated by volume-weighted averaging.

To assess differences in SUVRs between the VOI settings used for MR-based PVC, a repeated-measures analysis of variance (ANOVA) was performed for the whole cerebral cortex VOI and the SCWM VOI. Differences in SUVRs between MR-based PVC and PSF reconstruction were also analyzed using paired *t* tests. The significance level was set to *p* < 0.05.

## Results

### Reconstruction with and without PSF modeling

**Fig. 2:**
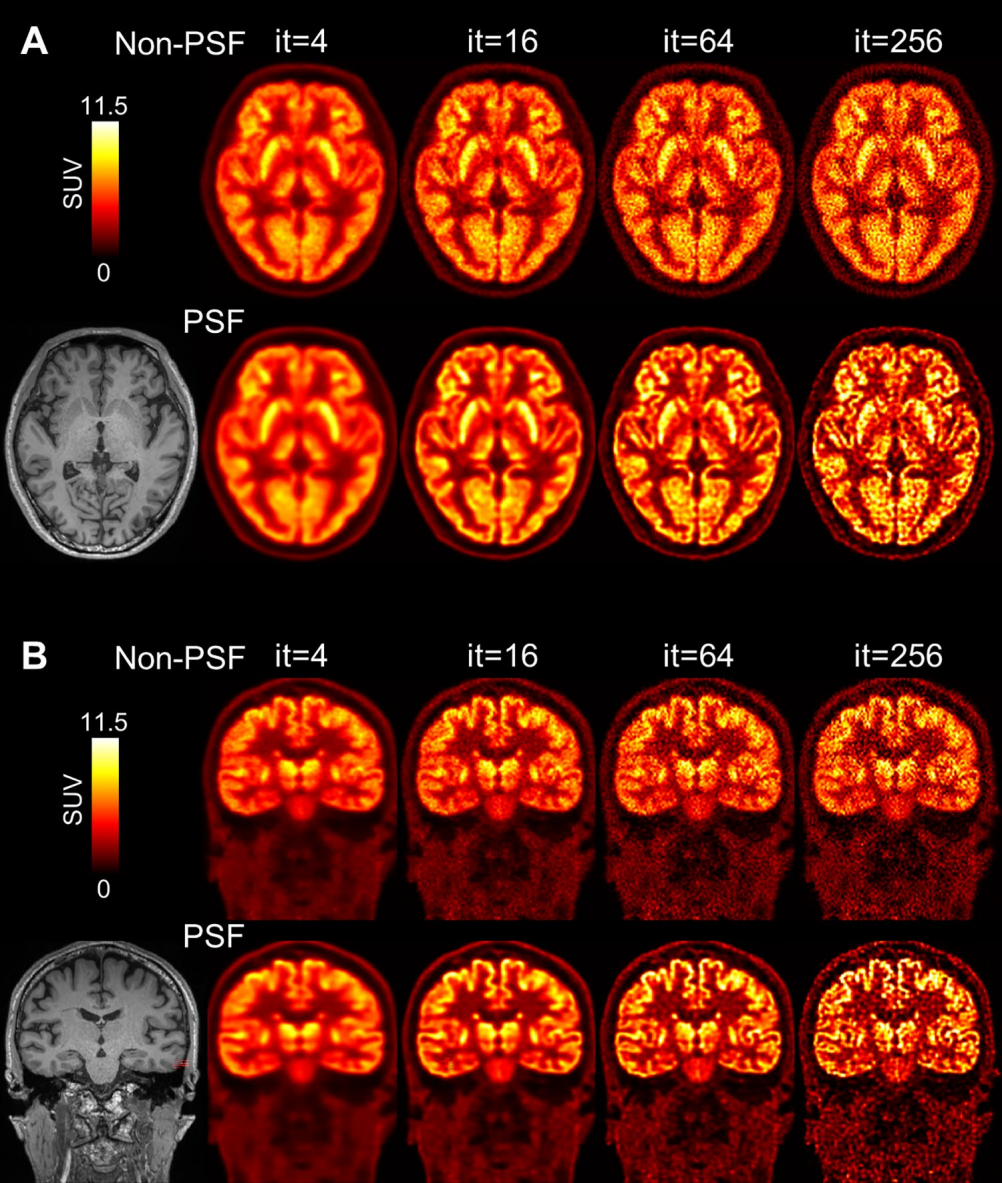
**A** Representative PET axial images (subject ID = 1; 59 y/male, 59 kg body weight) reconstructed without (upper) and with (lower) PSF modeling. The number of iterations ranges from 4 to 256. PET data were acquired with a Biograph Vision digital PET/CT system, 30 to 60 min (30-min scan duration) after intravenous injection of FDG (237 MBq). **B** Coronal PET images

**Fig. 3:**
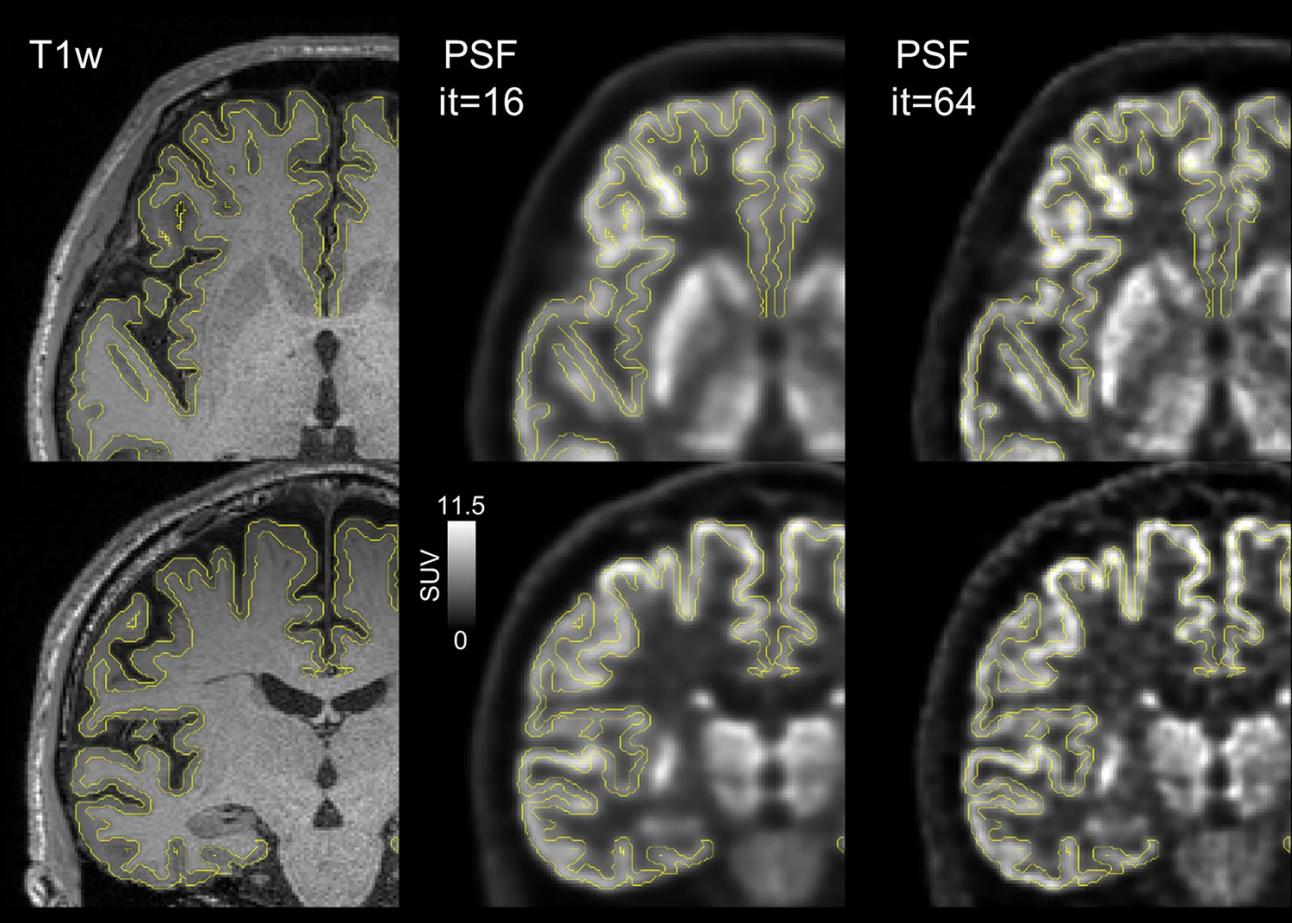
PSF reconstruction images (it = 16 and 64) and MR T1-weighted images from the same subject as in Fig. 2 (subject ID = 1). Overlaid yellow lines represent MR-segmented cerebral cortical boundaries (FreeSurfer)

Representative non-PSF and PSF reconstruction images are shown in Fig. 2. In the PSF reconstruction images, image contrast gradually increased with the increasing number of iterations. In contrast, in the non-PSF reconstruction images, the image contrast was relatively constant over all iterations. In Fig. 3, the MR-derived cortical boundaries (segmentation results from FreeSurfer) are overlaid on the PSF reconstruction images, demonstrating that the fine cortical structures that appeared in the PSF images are anatomically well matched with the MR-derived cortical segments. Reconstruction images for another subject are shown in Supplementary Figs. 3 and 4.

**Fig. 4:**
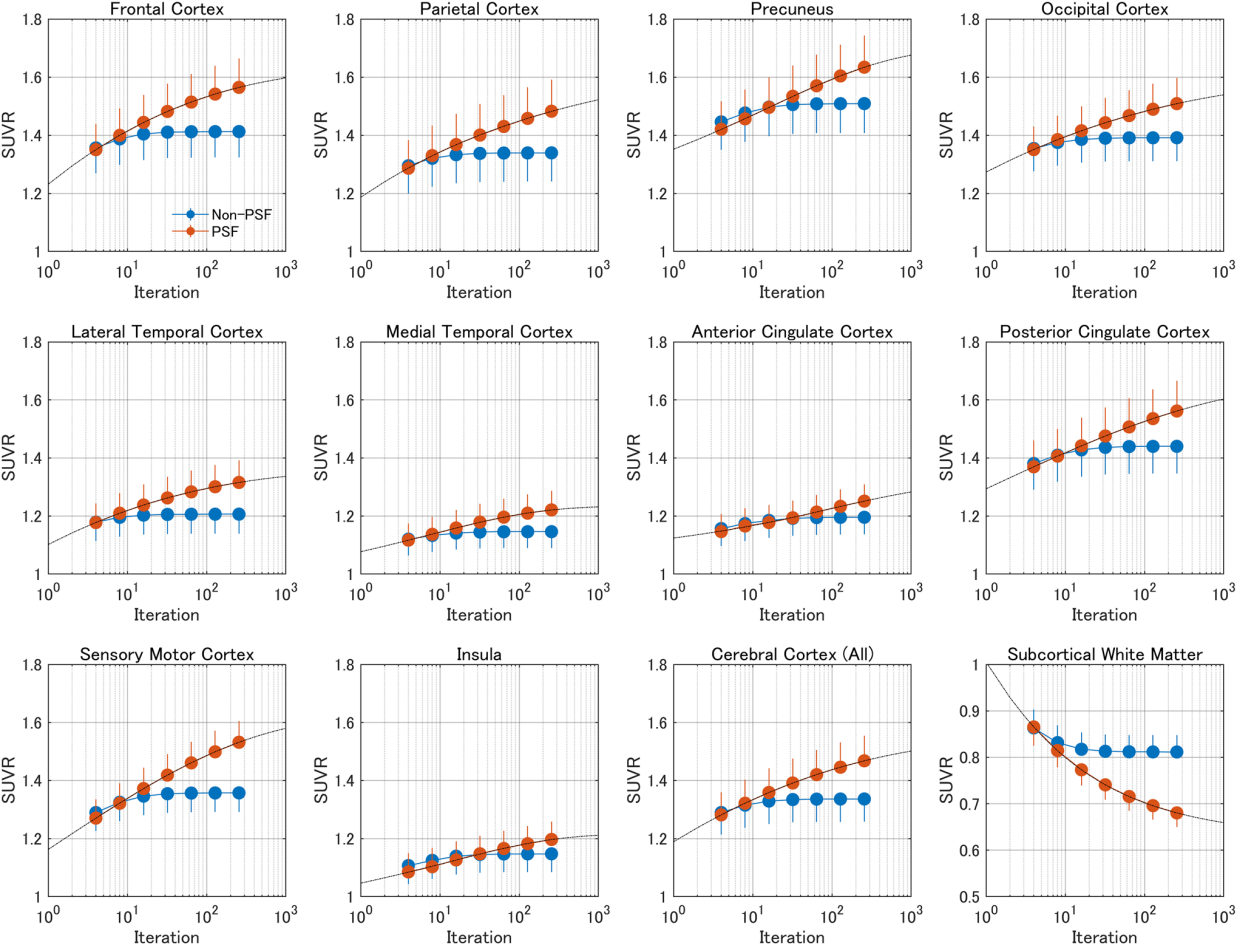
Mean and standard deviation of the standardized uptake value ratios (SUVRs) with non-PSF (blue) and PSF reconstruction (red) plotted against the number of iterations. SUVRs are from cortical regions and subcortical white matter (SCWM), calculated with the cerebellar cortex as a reference region. “Cerebral Cortex (All)” includes all cortical VOIs. Dashed-dotted lines represent fitted curves for PSF reconstruction

The mean SUVs (Supplementary Fig. 5) and SUVRs (Fig. 4) as a function of the number of iterations showed similar trends for all cerebral cortical regions. In the reference region (cerebellar cortex), as summarized in Supplementary Table 2, the mean SUV values were similar with both non-PSF and PSF reconstructions over a wide range of iterations because of relatively large volumes defined in FreeSurfer segmentation (Supplementary Fig. 1); therefore, we focused on SUVR as the index of FDG uptake. The mean SUVRs for the whole cerebral cortex and SCWM are summarized in Table 1. With the minimum number of iterations (it = 4), the mean SUVRs from non-PSF and PSF reconstructions were quite similar in both the whole cerebral cortex (1.29 vs 1.28, respectively) and SCWM (0.86 vs 0.87). Higher numbers of iterations resulted in differences between non-PSF and PSF reconstructions, with the mean SUVRs reaching 1.47 in the whole cerebral cortex and 0.68 in SCWM with the PSF reconstruction, but the values plateauing in the non-PSF images. The maximum number of iterations (it = 256) was insufficient for full convergence in the PSF images, as shown in Fig. 4. We thus extrapolated the mean SUVRs to an infinite number of iterations (it = ∞) by curve fitting with an empirical exponential-type function:

$${SUVR}_{PSF}=a\times \left[1-{e}^{-b\times {IT}^{c}}\right]+d,$$

where *IT* indicates the number of iterations in PSF reconstruction and *a*, *b*, *c*, and *d* are free parameters. The fitted curves are shown in Fig. 4 as dashed-dotted lines. The mean SUVRs for an extrapolated infinite number of iterations were 1.57 for the whole cerebral cortex (7% higher than the value for 256 iterations) and 0.63 for SCWM (7% lower than the value for 256 iterations; Table 1).

**Table 1:** Standardized uptake value ratios (SUVRs; mean ± standard deviation) in the cerebral cortex and subcortical white matter (SCWM)

	a) Reconstruction: 3D OP-OSEM TOF			b) MR-based PVC	
	Iteration	Non-PSF	PSF	4.0-mm FWHM	3.5-mm FWHM
Cerebral cortex	4	1.29 ± 0.08	1.28 ± 0.08	1.76 ± 0.11 [1.74, 1.81]	1.70 ± 0.11 [1.67, 1.75]
	16	1.33 ± 0.08	1.36 ± 0.08		
	64	1.34 ± 0.08	1.42 ± 0.09		
	256	1.34 ± 0.08	1.47 ± 0.09		
	∞	NA	1.57 ± 0.12		
SCWM	4	0.86 ± 0.04	0.87 ± 0.04	0.50 ± 0.02 [0.47, 0.52]	0.54 ± 0.02 [0.51, 0.58]
	16	0.82 ± 0.04	0.77 ± 0.03		
	64	0.81 ± 0.04	0.72 ± 0.03		
	256	0.81 ± 0.04	0.68 ± 0.03		
	∞	NA	0.63 ± 0.04		

SUVRs were calculated with the cerebellar cortex as a reference region. Values in parentheses represent minimum and maximum of the mean SUVRs among five VOI settings in the MR-based PVC

The VOI analysis also showed that the mean CoV values increased with the increasing number of iterations for both non-PSF and PSF reconstructions (Supplementary Fig. 6).

### MR-based PVC

**Fig. 5:**
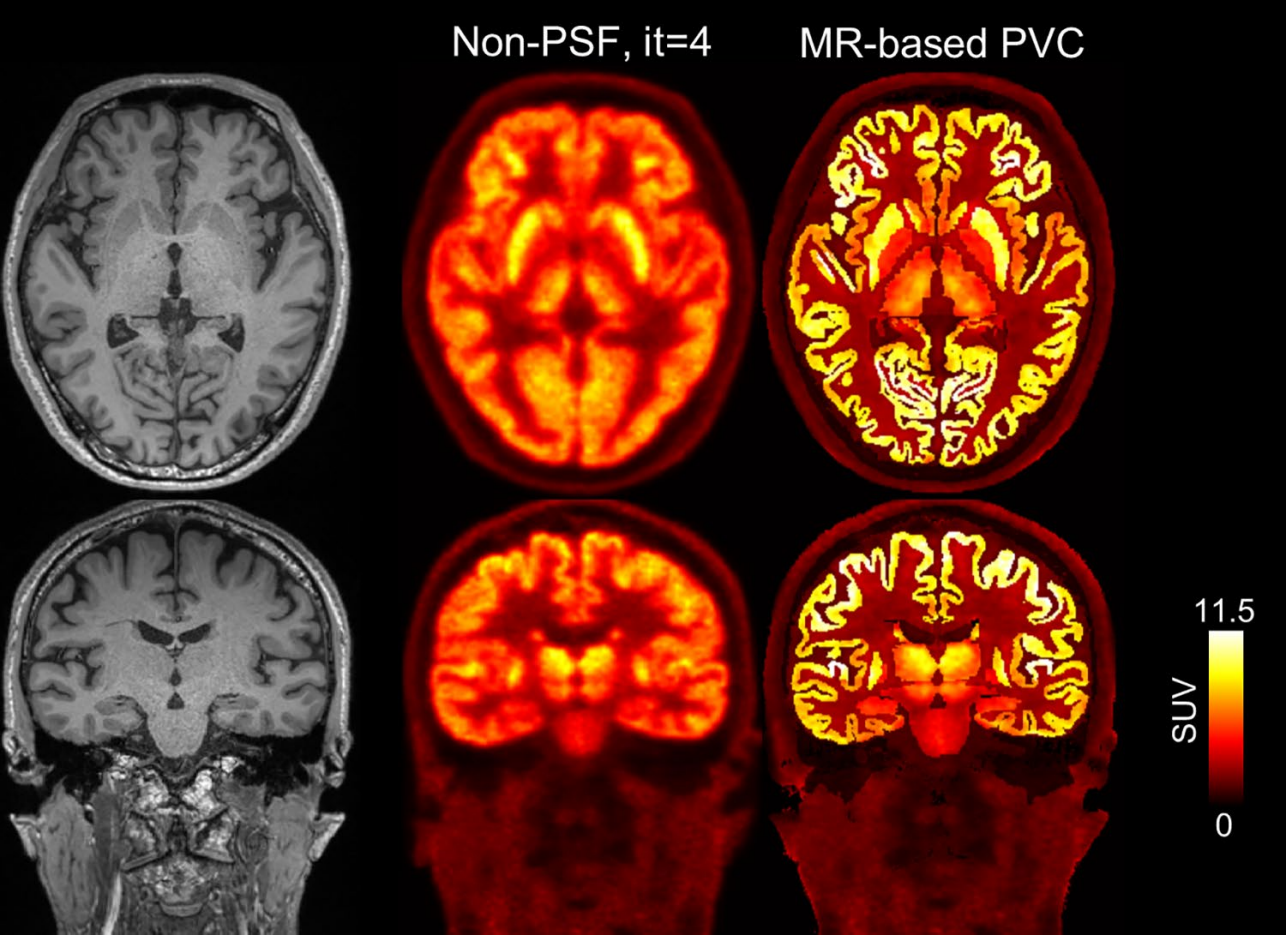
Representative partial volume corrected (PVC) PET maps (subject ID = 1) processed with region-based voxel-wise correction (RBV) and a 4.0-mm FWHM spatial resolution kernel

Representative PVC maps are shown in Fig. 5. In the cerebral cortex, the PVC maps showed clearly higher signals with more uniform distribution than the input images (non-PSF images). Although these PVC maps were based on the *VOI*_*Full*_ setting (the reference condition), we present other maps in Supplementary Fig. 7.

The mean SUVRs of the MR-based PVC with the reference condition (*VOI*_*Full*_ setting) in the whole cerebral cortex and SCWM are summarized in Table 1, with values in parentheses representing the minimum and maximum of the mean SUVRs among the five VOI settings. The complete results of the MR-based PVC are provided in Supplementary Table 3. The ANOVA showed significant differences between the VOI settings (*p* < 1e-19). In the whole cerebral cortex, the mean SUVRs ranged from 1.67 (3.5-mm FWHM; *VOI*_*Full*+*WM2*_) to 1.81 (4.0-mm FWHM; *VOI*_*BG*_), and were significantly higher than the SUVRs from the PSF reconstruction with infinite extrapolation (1.67 with MR-based PVC vs. 1.57 with PSF reconstruction; *p* < 1e-4). In SCWM, the mean SUVRs ranged from 0.47 (4.0-mm FWHM; *VOI*_*BG*_) to 0.58 (3.5-mm FWHM; *VOI*_*Full*+*WM2*_), and were significantly lower than the SUVRs from the PSF reconstruction with infinite extrapolation (0.58 in MR-based PVC vs. 0.63 in PSF reconstruction; *p* < 1e-6).

## Discussion

Cortical structures were clearly depicted by the combination of PSF reconstruction and the latest digital PET scanner. The main finding from the quantitative evaluation is that higher numbers of iterations resulted in higher cortical SUVRs, which approached the values of the SUVRs of the MR-based PVC, but were still smaller by 16%, even with the maximum number of iterations (it = 256). This inter-method difference between PSF reconstruction and MR-based PVC could not be explained by variations in the VOI and FWHM settings in the MR-based PVC.

### PSF reconstruction as a PVC method in brain PET

Slow convergence in PSF reconstruction is well known and theoretical and experimental investigations have been performed (e.g., [21]); however, reporting count recovery curves with a sufficiently wide range of iterations is very rare with actual brain PET data, and the present study is the first demonstration for the current PET scanner. With the minimum number of iterations evaluated (it = 4), which corresponds to the setting for clinical whole-body ^18^F-FDG, both PSF and non-PSF reconstructions showed similar image contrast and SUVRs, meaning that the merits of PSF reconstruction with a low number of iterations do not include higher image contrast, but rather a smoother texture with lower noise. To gain the greatest benefit of PSF reconstruction, namely higher image contrast that is unachievable by non-PSF reconstruction, we recommend at least it = 16 for brain scanning with the current PET scanner (Biograph Vision) and the reconstruction implementation (3D OP-OSEM with TOF and PSF; 5 subsets). The upper limit for iterations should be determined in the context of an acceptable noise level for a specific application.

Because of its slow convergence characteristic [21], the SUVRs from PSF reconstruction did not converge sufficiently, even with the maximum number of iterations (it = 256), remaining 16% smaller than the corresponding values for MR-based PVC in the cerebral cortex (1.47 vs. 1.76). The curve fitting analysis further demonstrated that even if the number of iterations in PSF reconstruction is increased to an infinite amount (it = ∞), cortical SUVRs cannot reach the values from MR-based PVC, remaining 11% lower in the cerebral cortex (1.57 vs. 1.76). This implies the incomplete resolution recovery in PSF reconstruction. To the best of our knowledge, this is the first study that evidenced the incomplete recovery in PSF reconstruction with ideal settings (i.e., sufficiently large number of iterations and the extrapolation analysis) based on actually acquired PET data (not simulation data), which is not surprising given that the high spatial frequency components are already partially lost during the PET acquisition [9, 10, 31] and are thus difficult to recover by any PET-alone image-processing technique.

The extent of the effective spatial resolution reached with PSF reconstruction can be estimated by a numerical simulation under the assumption that MR-based PVC maps represent the true tracer distribution. Supplementary Fig. 8 shows simulated cortical SUVR estimates as a function of effective spatial resolution, with these being derived using Gaussian-smoothed MR-based PVC maps as ground-truth data. According to these results, the bias observed in the present study, − 16% with the maximum number of iterations (it = 256), corresponds to approximately 1.5-mm FWHM, equivalent to the effective spatial resolution reported with the HRRT scanner and PSF reconstruction (16 subsets and 6 iterations), where an FWHM of 1.4 mm was derived from a point source experiment [32].

One criticism of PSF reconstruction is edge artifacts, a rippling phenomenon visible at the edges of the uptake regions [31, 33, 34]. It is not easy to predict the extent and strength of such edge artifacts because they depend on complicated interactions between various factors including the physical dimensions of the object (size, shape), PSF, and number of iterations. In the present study, remarkable edge artifacts were not noted, at least in cerebral cortical regions (Fig. 3). The cerebral cortex is quite thin, typically being less than 3 mm [3], and sharp edges on both sides of the cortex may be overlapping and hidden [31]. Although the impact is minimal for average values inside VOIs that are independently derived from an MR image as in the present study, the edge artifacts can cause serious overestimation of SUV_max_ (maximum within a VOI) [35], cautioning the use of PSF reconstruction in such applications.

### Limitations

This study compared PSF reconstruction and MR-based PVC in a small number of healthy subjects; it did not determine which approach is better for a particular clinical task. The acceptable level of image noise depends on the specific PET application; the optimal number of iterations for a particular application cannot be derived from the present results. In addition, inter-subject variability and within-subject reproducibility, which are important considerations in clinical applications, were not assessed. Rather, the purpose of this study was to clarify the maximum performance of PSF reconstruction when used as a PVC method. The current implementation of PSF reconstruction is the most standard and straightforward, but more advanced algorithms such as Bayesian penalized likelihood reconstruction have better noise-contrast characteristics [36] and may outperform in terms of inter-subject variability and reproducibility, warranting future investigation.

This comparative study used MR-based PVC as the reference; however, it should be remembered that MR-based PVC relies on the two assumptions, spatial uniformity of tracer uptake within predefined VOIs and exactly known effective spatial resolution of input images, and thus is not the gold standard. Therefore, although the present study showed lower SUVR estimates with PSF reconstruction compared to MR-based PVC irrespective of variations in the VOI and FWHM settings (Table 1), the degree of underestimation, that is − 16% with the maximum number of iterations (it = 256), should not be regarded as a true bias. As demonstrated by the phantom experiments (Supplementary Fig. 2), differences in the diameter of the spheres and signal-to-background ratio resulted in different estimates of effective spatial resolution; the nature of object-dependent recovery in OSEM reconstruction may be a significant limitation of MR-based PVC. In addition to the reference condition (4.0-mm FWHM), the current MR-based PVC applied 3.5-mm FWHM, which is in line with the lower bound of estimated FWHMs for non-PSF reconstruction (it = 4; Supplementary Fig. 2). The analysis showed that the cortical SUVRs were still smaller with PSF reconstruction (Table 1), indicating that this issue could not change the current conclusion. Nevertheless, the complex geometry of cerebral cortex, far from the spherical shape, makes estimation difficult, which warrants further examination.

### Further applications of PSF reconstruction

**Fig. 6:**
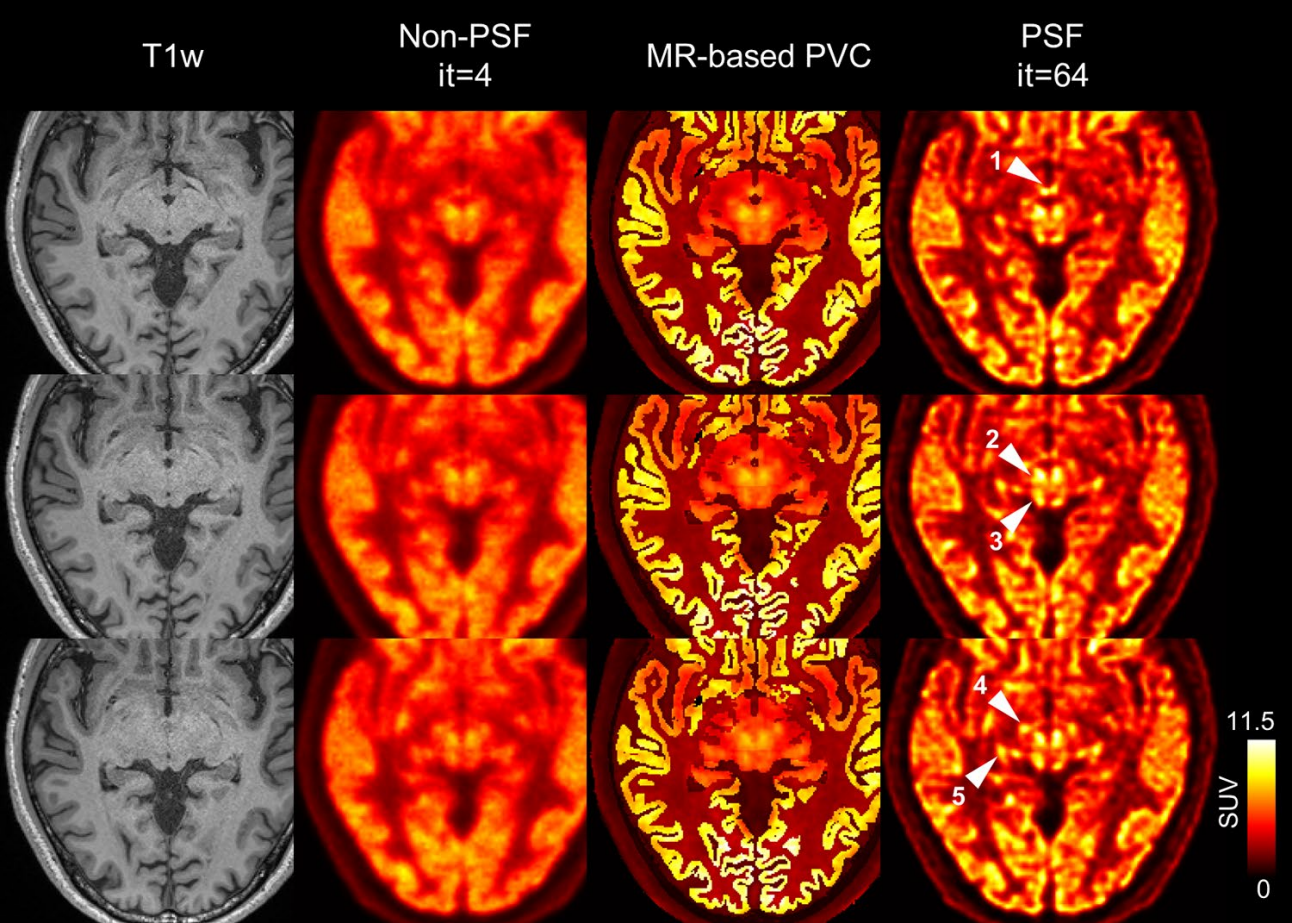
Three representative sequential slices exhibit high uptake of FDG in several small structures (subject ID = 1). Left-to-right: T1w, non-PSF (it = 4), MR-based PVC, and PSF (it = 64). In contrast to PSF reconstruction, MR-based PVC shows weaker uptake in small structures. White arrowheads indicate small structures: (1) mammillary body; (2) red nucleus; (3) superior colliculus; (4) subthalamic nucleus; (5) lateral geniculate nucleus

Although its resolution recovery performance is not ideal when used as a PVC method, PSF reconstruction has the advantage that it does not depend on the assumptions required for MR-based PVC. The problem of VOI settings in MR-based PVC is of poor quality outside the cerebral cortex. Figure 6 compares PSF reconstruction and MR-based PVC in representative axial slices at the brainstem level. In the PSF reconstructions, microstructures with high FDG uptake (presumably corresponding to small nuclei) are clearly visible, but they are not noticeable on the MR-based PVC. This is because these microstructures are not visible on the T1-weighted images (and thus their segmented images), and therefore the MR-based PVC is ineffective. The latest digital PET combined with PSF reconstruction clearly depicts small brain nuclei, which was not considered possible with conventional clinical PET scanners, and presents an interesting research topic for future study [37–39].

## Conclusion

With the latest digital PET scanner, which has improved spatial resolution and sensitivity, PSF reconstruction can be used as a PVC technique in brain PET, albeit with suboptimal resolution recovery. The present study showed a 16% underestimation of ^18^F-FDG uptake in the cerebral cortex, even after a large number of iterations (it = 256), which corresponded to an effective spatial resolution of approximately 1.5-mm FWHM. A relative advantage of PSF reconstruction is its applicability to not only cerebral cortical regions, but also to various small structures such as small brain nuclei, which have never been investigated with conventional PET systems and present an interesting future research topic.

## Supplementary Information

Below is the link to the electronic supplementary material.

Supplementary file1 (DOCX 7872 KB)
