# Supplementary material for: Brain partial volume correction with point spreading function reconstruction in high-resolution digital PET: comparison with an MR-based method in FDG imaging
Source: Ann Nucl Med. 2022 May 26;36(8):717–27. doi: 10.1007/s12149-022-01753-5 (PMC9304042; doi:10.1007/s12149-022-01753-5)
Supplement: Supplementary file 1 — Supplementary file1 (DOCX 7872 KB) [file 12149_2022_1753_MOESM1_ESM.docx]

# Different VOI settings used in PVC

Supplementary Table 1 summarizes the VOI settings investigated. In the *VOI_BG_* setting the 111 brain regions (FreeSurfer segments including 68 cortical labels) plus a non-brain background (all voxels outside the brain) resulted in a total of 112 regions, which were adopted as the VOIs for PVC calculation. In the *VOI_Outer_* setting, a more realistic non-brain region was defined by dilating the brain tissue image, resulting in a 15-mm shell surrounding the outer surface of the brain, which was used as the background region in the PVC calculation (Matsubara et al. 2020). The *VOI_Full_* setting adopted individual extracerebral segmentations (Greve et al. 2016) derived from tissue segmentation performed on individual T1-weighted images using the SPM segmentation tool. The *VOI_Full_* setting was regarded as the reference setting in the present study. In the aforementioned VOI settings subcortical white matter (SCWM) was treated as a single large segment in each hemisphere. In the *VOI_Full+WM_* setting, the SCWM region in each hemisphere of each subject was further segmented into 35 anatomical regions using FreeSurfer’s white matter parcellation (*wmparc.mgz*), resulting in a total of 182 regions. In the *VOI_Full+WM2_* setting, each small SCWM VOI (35 VOIs in a hemisphere) was split into two layers corresponding to deep SCWM and surface SCWM in the vicinity of the cerebral cortex. The cortical label images were dilated with a 3 × 3 × 3-voxel kernel, and overlapping areas in the original SCWM were generated as the surface SCWM regions comprising new 70 WM segments (in each hemisphere), resulting in a total of 252 regions. Supplementary Figure 1 shows representative label images for each VOI setting.

**Supplementary Table 1:** Summary of the different VOI settings in the MR-based PVC


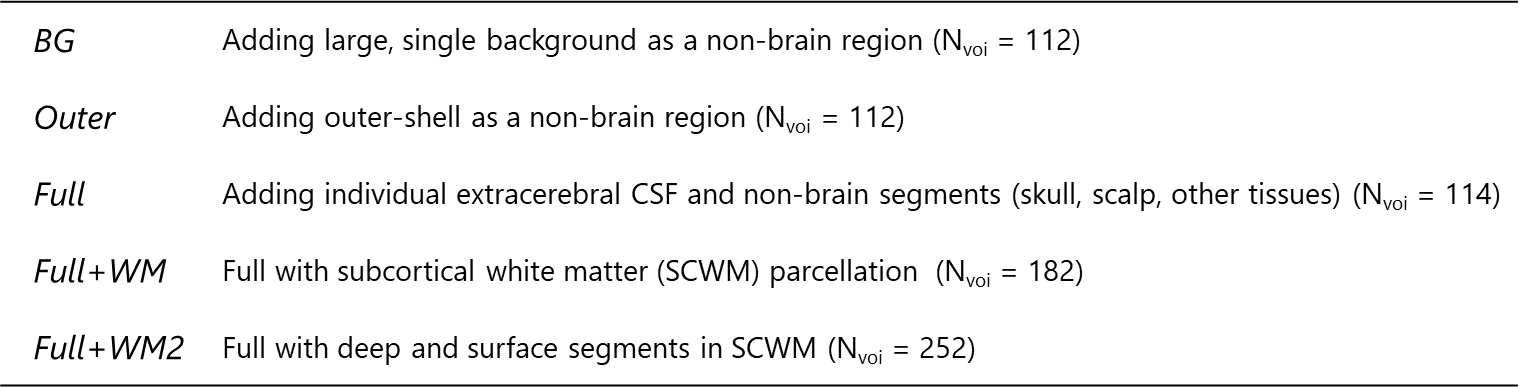


**
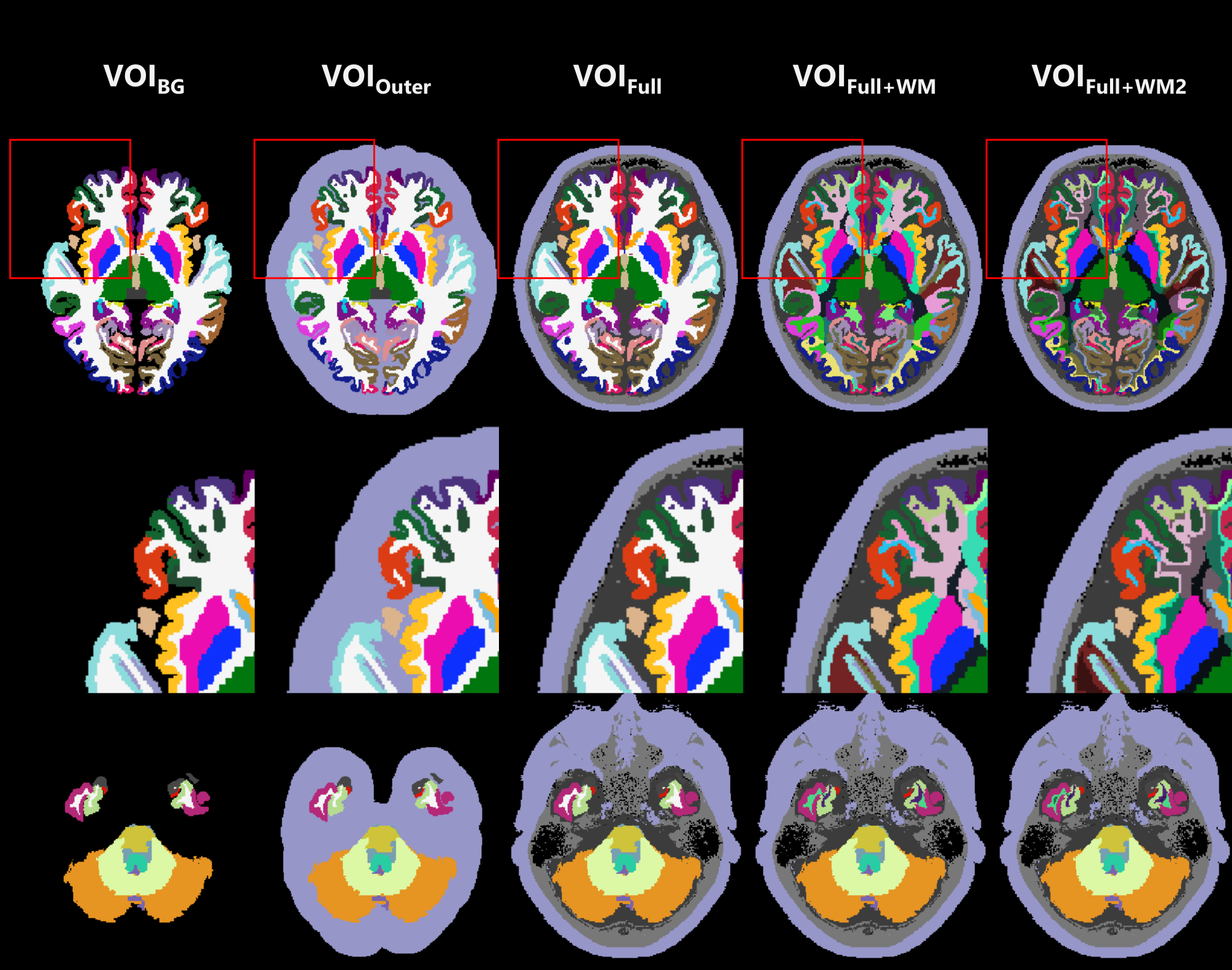
 Supplementary Figure 1:** Volume of interest settings for a subject (ID=1) used for the PVC: basal ganglia level (top), zoomed version (middle), and cerebellar level (bottom).

# Phantom experiments for estimating effective spatial resolution in PET reconstruction images

Phantom experiments were performed to estimate the effective spatial resolution of reconstructed PET images. A cylinder phantom (200 mm in diameter, 185 mm in length) with six fillable hot-spheres with diameters ranging from 5.0 to 38.0 mm was scanned three times with different signal-to-background ratios: 4:1, 8:1, and 16:1 (2.6 kBq/mL of ^18^F solution in the background). The scan duration in each experiment was 60 minutes, resulting in high quality images with sufficiently high statistics. Image reconstruction was performed with settings identical to those used in the human brain study. The effective resolution was estimated using the profile fitting method (Hofheinz et al. 2010). In this method, profile curves as a function of distance from the center position of each hot-sphere were fitted with an analytical function with three parameters: spatial resolution (FWHM), sphere diameter, and signal-to-background ratio. The cold walls of the hot sphere (1-mm fixed) were included in the model equation. Results of the FWHM estimates as a function of reconstruction iterations are shown in Supplementary Figure 2 (lower left). For non-PSF with 4 iterations (input to MR-based PVC), FWHM estimates were 3.5 to 4.5 mm, depending on the sphere diameters and signal-to-background ratios. Based on these results, a 4.0 mm FWHM was chosen for the PSF in the MR-based PVC.

**Supplementary Figure 2:** Results of the phantom experiments. PET images were reconstructed with non-PSF (left column) and PSF (right column). Upper row: recovery (%) of signal-to-background ratio (SBR), defined as measured SBR divided by the true SBR (from known activity concentrations). Lower row: full width at half maximum (FWHM) of Gaussian PSF kernels, estimated using the profile fitting method (Hofheinz et al). Different colors correspond to diameters of the hot spheres (“D” in figure panels), and line types to SBRs in the experiments: 4 (solid lines), 8 (dashed-dotted lines), and 16 (dotted lines).


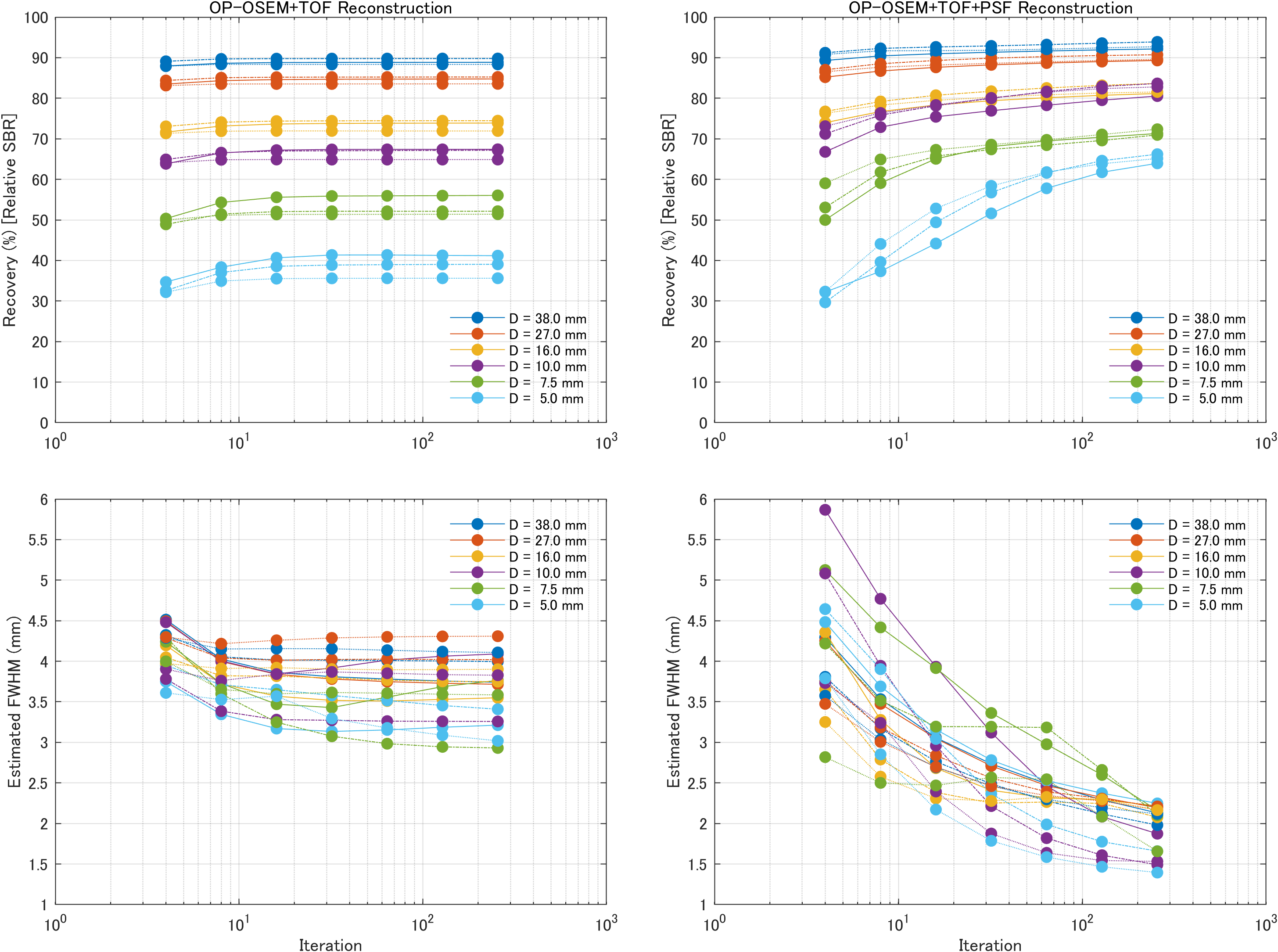


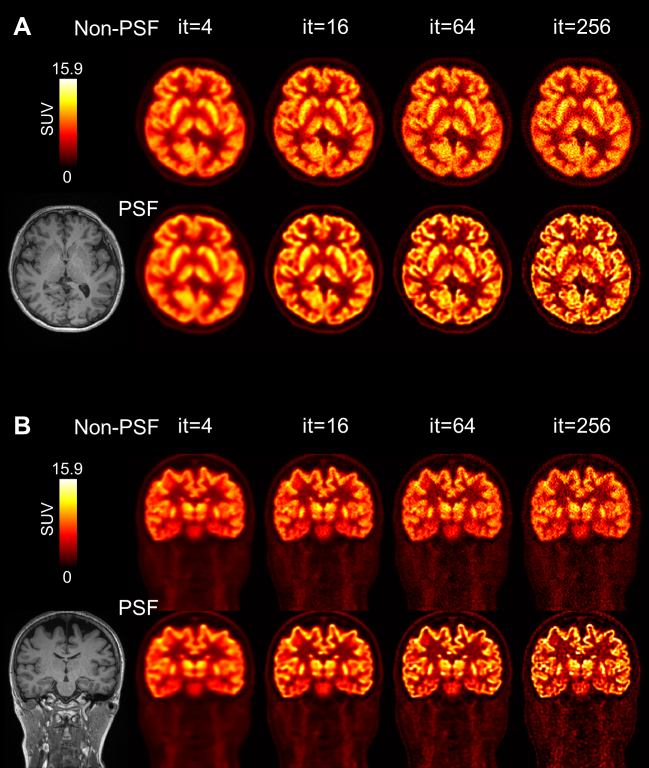
**Supplementary Figure 3:** Similar to Figure 2 but from another subject (ID=4; 60 years/female, 40 kg body weight; 224 MBq of FDG injected).

**
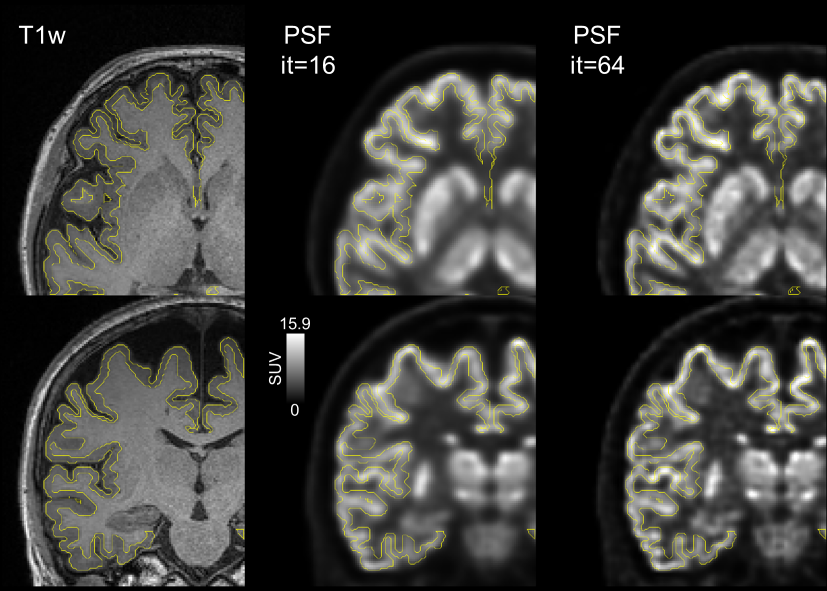
Supplementary Figure 4:** Similar to Figure 3 but from another subject (ID=4; 60 years/female, 40 kg body weight; 224 MBq of FDG injected).


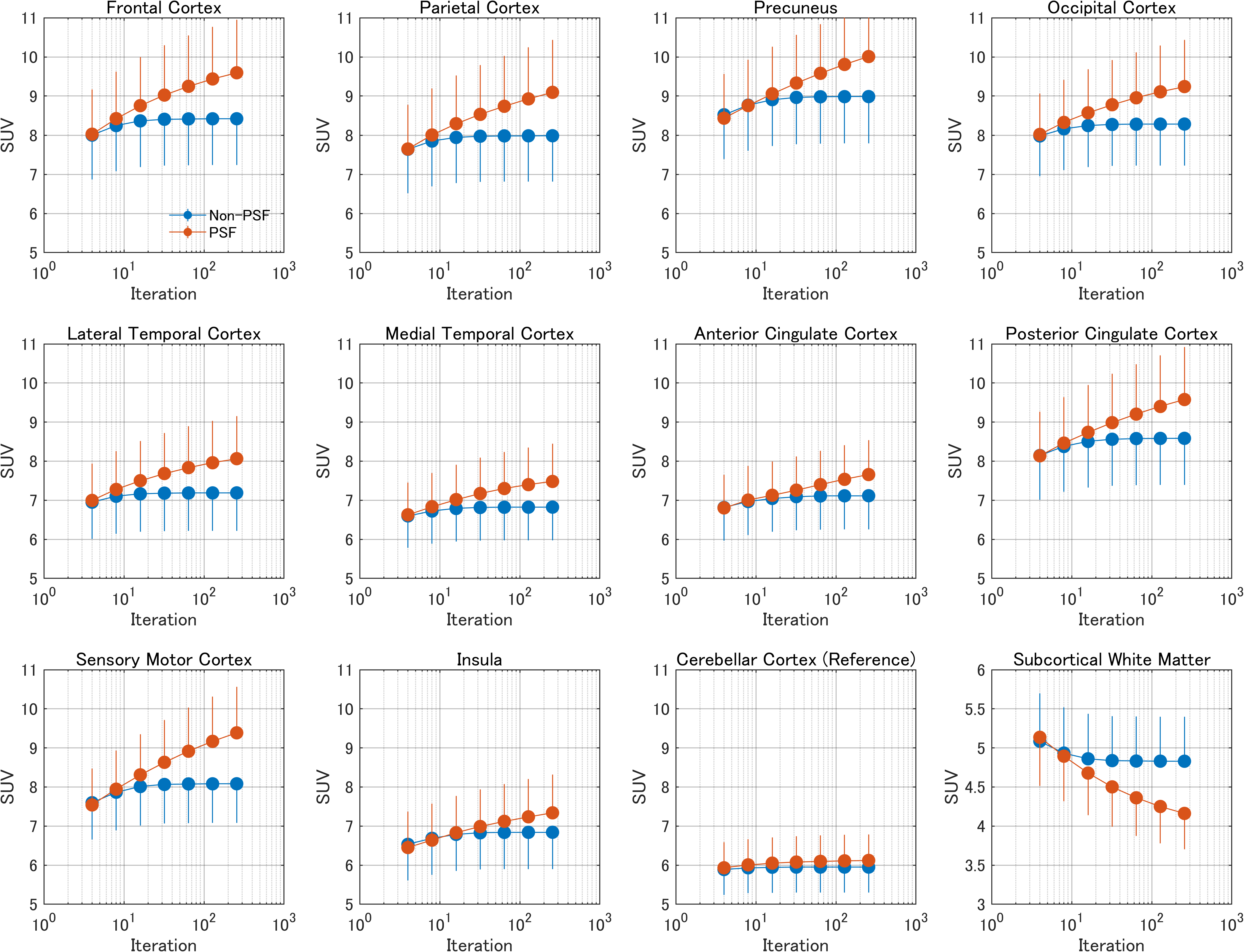


**Supplementary Figure 5:** Mean and standard deviation of the standardized uptake value (SUVRs) with non-PSF (blue) and PSF reconstruction (red) plotted against number of iterations. SUVs are from cortical regions, cerebellar cortex (reference region), and subcortical white matter (SCWM).

**Supplementary Table 2:** Standardized uptake value (SUVs; mean ± standard deviation) in cerebral cortex, subcortical white matter (SCWM), and cerebellar cortex (reference region).

|  | a) Reconstruction: 3D OP-OSEM TOF | | | b) MR-based PVC | |
| --- | --- | --- | --- | --- | --- |
|  | Iteration | Non-PSF | PSF | 4.0-mm FWHM | 3.5-mm FWHM |
| Cerebral Cortex | 4 | 7.61 ± 1.04 | 7.61 ± 1.04 | 11.1 ± 1.54  [11.0, 11.6] | 10.6 ± 1.46  [10.4, 11.2] |
|  | 16 | 7.92 ± 1.07 | 8.24 ± 1.13 |  |  |
|  | 64 | 7.96 ± 1.08 | 8.67 ± 1.18 |  |  |
|  | 256 | 7.96 ± 1.08 | 9.00 ± 1.23 |  |  |
| SCWM | 4 | 5.09 ± 0.61 | 5.14 ± 0.62 | 3.14 ± 0.31  [3.02, 3.27] | 3.37 ± 0.34  [3.24, 3.59] |
|  | 16 | 4.86 ± 0.57 | 4.68 ± 0.54 |  |  |
|  | 64 | 4.83 ± 0.57 | 4.36 ± 0.49 |  |  |
|  | 256 | 4.83 ± 0.57 | 4.16 ± 0.46 |  |  |
| Cerebellar Cortex | 4 | 5.89 ± 0.65 | 5.93 ± 0.66 | 6.29 ± 0.69  [6.29, 6.41] | 6.22 ± 0.68  [6.10, 6.33] |
|  | 16 | 5.95 ± 0.65 | 6.05 ± 0.66 |  |  |
|  | 64 | 5.95 ± 0.65 | 6.10 ± 0.66 |  |  |
|  | 256 | 5.95 ± 0.65 | 6.12 ± 0.67 |  |  |


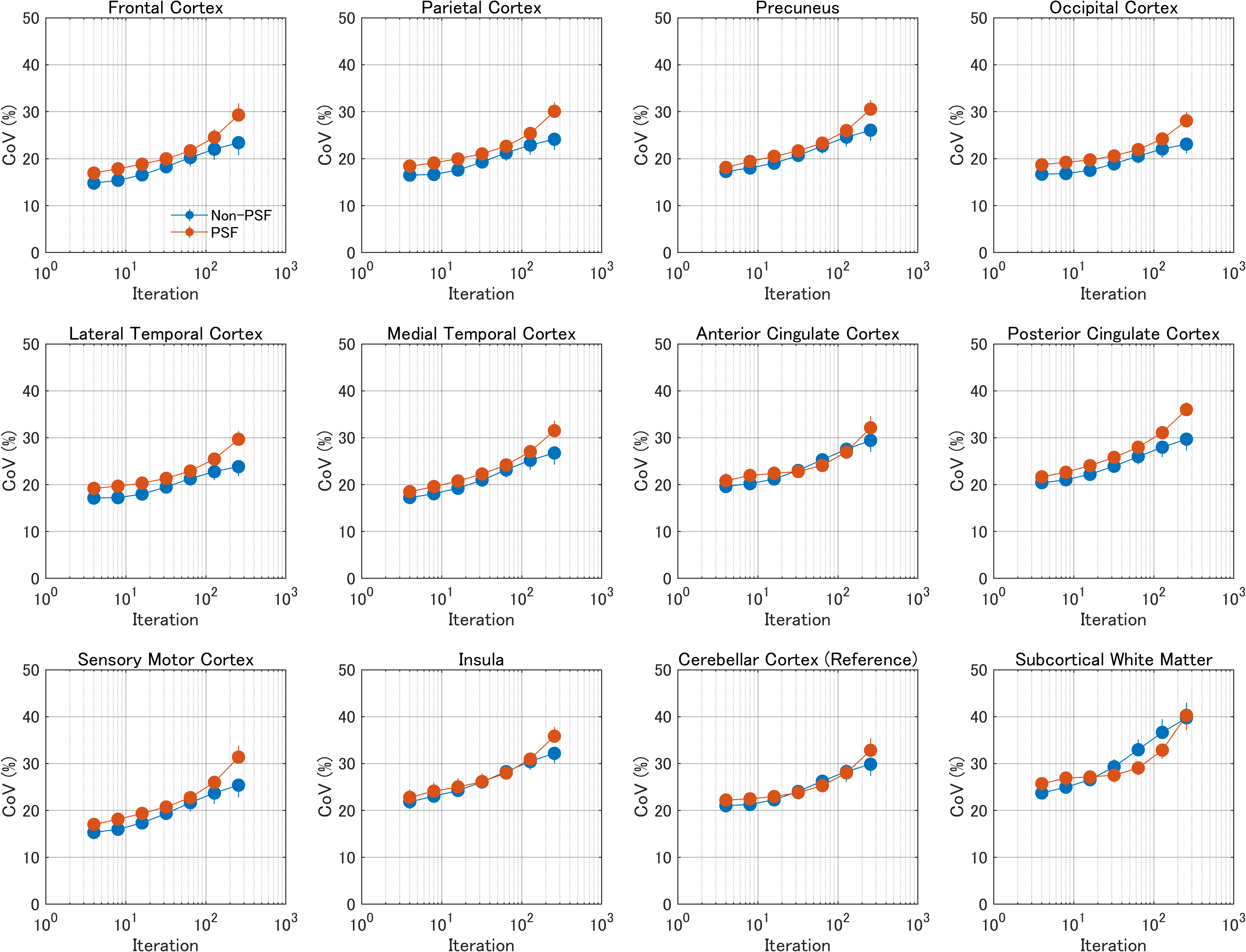


**Supplementary Figure 6:** Intra-region coefficient of variation (CoV; standard deviation in VOI divided by the mean in % unit) with non-PSF (blue) and PSF reconstruction (red) plotted against number of iterations.


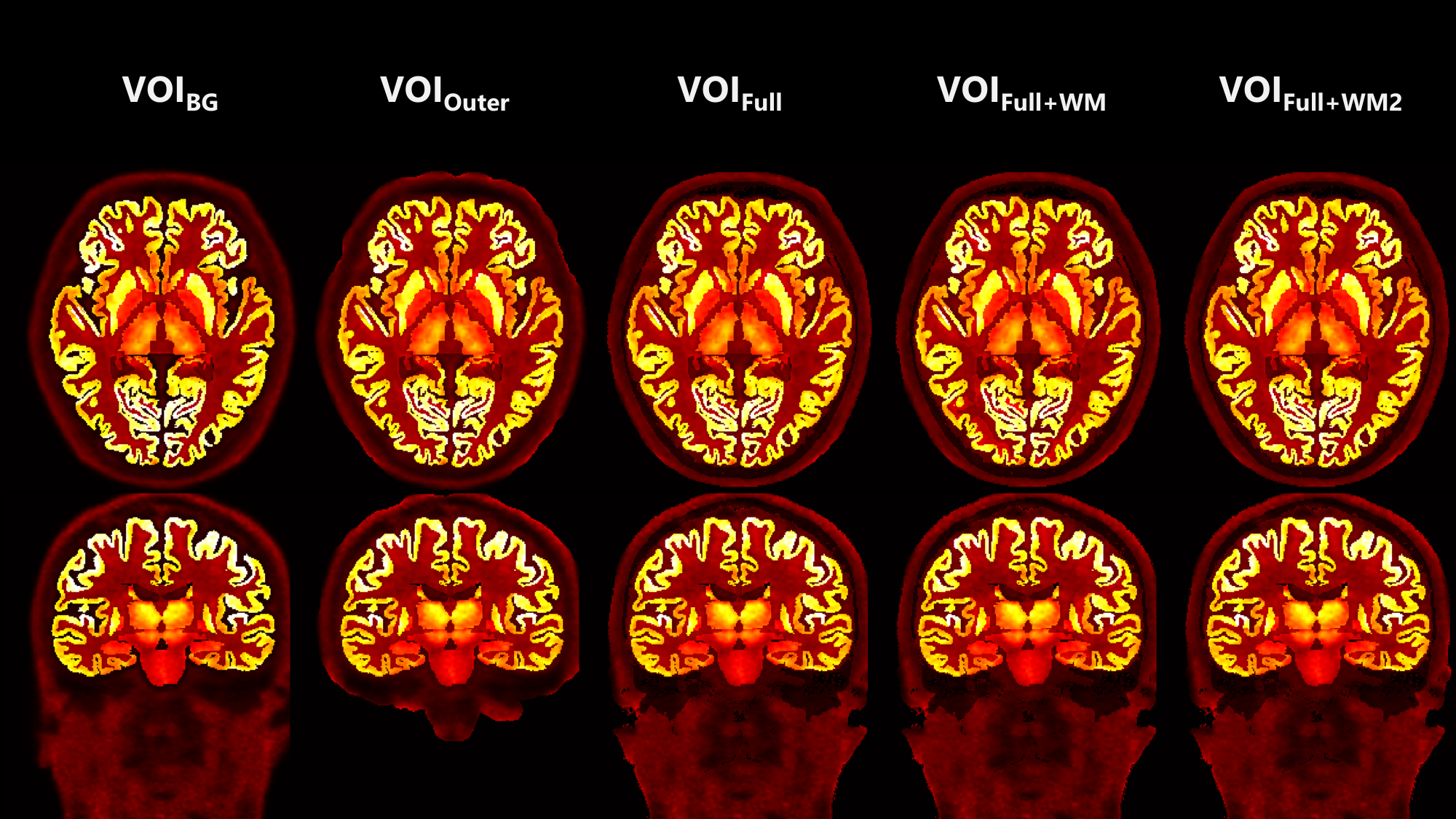
**Supplementary Figure 7:** Partial volume corrected (PVC) PET maps (subject ID=1) processed with a region-based voxel-wise correction (RBV), with various volume of interest (VOI) settings for PVC (4.0 mm FWHM).

**Supplementary Table 3:** Standardized uptake value ratios (SUVRs; averages ± standard) from MR-based partial volume correction (PVC) and region-based voxel-wise correction (RBV) for cerebral cortex volumes-of-interest (VOIs) (including all cortical VOIs) and SCWM. SUVRs were calculated with cerebellar cortex as a reference region. Values in % are differences from the reference setting (VOI “Full” and 4.0-mm FWHM).

| MR-based PVC | VOI | 4.0-mm FWHM | | 3.5-mm FWHM | |
| --- | --- | --- | --- | --- | --- |
| Cerebral Cortex | BG | 1.81 ± 0.11 | +2% | 1.75 ± 0.11 | -1% |
|  | Outer | 1.79 ± 0.11 | +1% | 1.74 ± 0.11 | -2% |
|  | Full | 1.76 ± 0.11 | Reference | 1.70 ± 0.11 | -3% |
|  | Full+WM | 1.75 ± 0.11 | -1% | 1.69 ± 0.11 | -4% |
|  | Full+WM2 | 1.74 ± 0.11 | -1% | 1.67 ± 0.10 | -5% |
| SCWM | BG | 0.47 ± 0.02 | -6% | 0.51 ± 0.02 | +2% |
|  | Outer | 0.49 ± 0.02 | -2% | 0.53 ± 0.02 | +6% |
|  | Full | 0.50 ± 0.02 | Reference | 0.54 ± 0.02 | +9% |
|  | Full+WM | 0.51 ± 0.02 | +2% | 0.56 ± 0.02 | +11% |
|  | Full+WM2 | 0.52 ± 0.03 | +4% | 0.58 ± 0.03 | +16% |

**
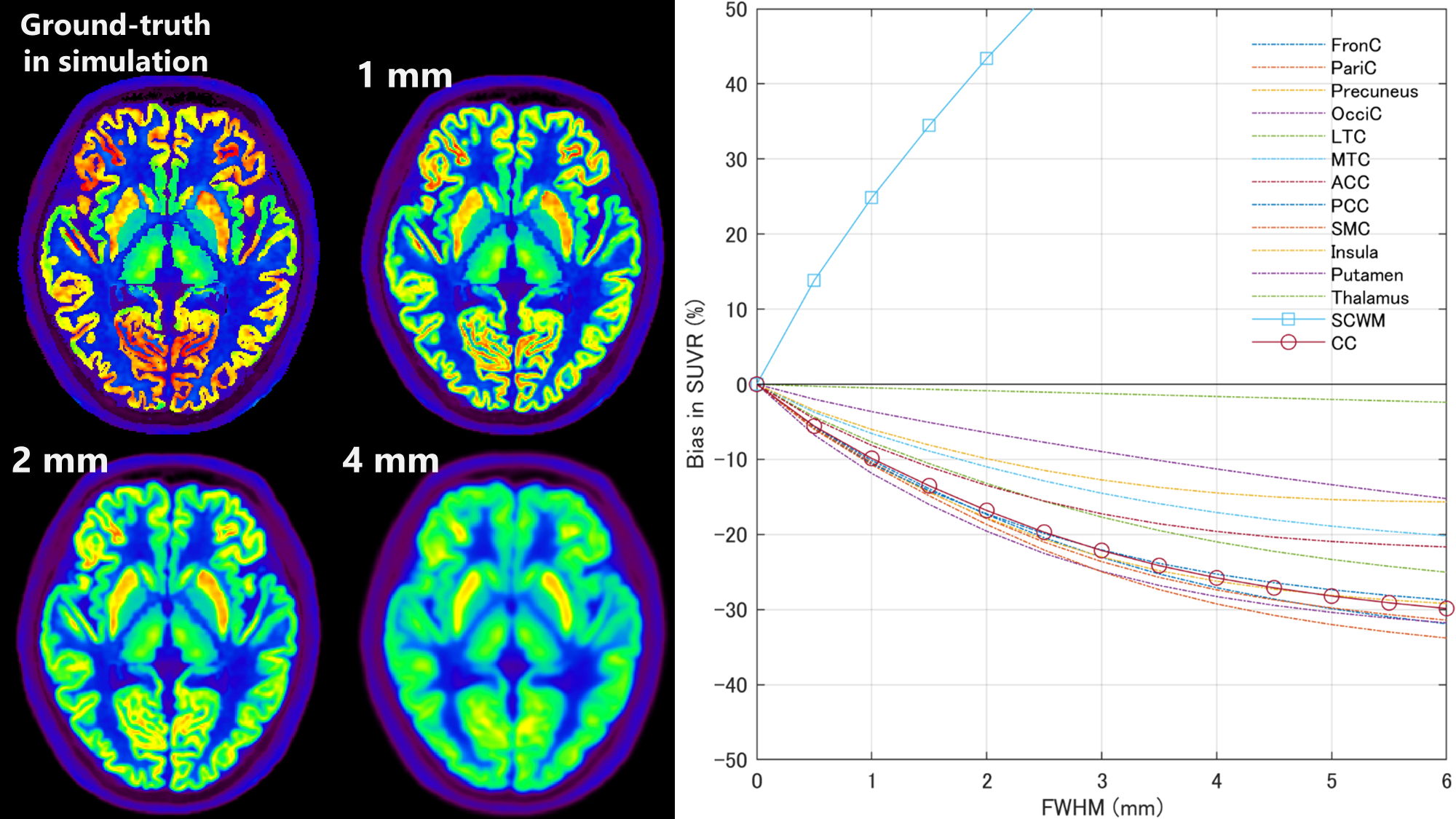
Supplementary Figure 8:** Simulation of SUVR bias as a function of effective spatial resolution (FWHM [mm]) in reconstruction images. Tracer uptake distribution (ground-truth) was assumed to be identical with that in RBV maps (*VOI_Full_*). The estimated biases presented here (right panel) are averaged over all subjects (n=10). The representative ground-truth image and smoothed versions are from Subject 1 (left panel).
